# The effect of empowerment program to reduce Sugar Consumption based on the Multi-Theory Model on Body Mass Index and abdominal obesity in Iranian women

**DOI:** 10.1186/s12905-023-02361-9

**Published:** 2023-04-28

**Authors:** Hamid Joveini, Nader Sharifi, Batool Kalate Meymary, Ali Mehri, Reza Shahrabadi, Vahid Rahmanian, Masoumeh Hashemian

**Affiliations:** 1 Department of Health Education and Health Promotion, Faculty of Health, Sabzevar University of Medical Sciences, Sabzevar, Iran; 2 Department of Public Health, Khomein University of Medical Sciences, Khomein, Iran; 3 Department of Public Health, Torbat Jam Faculty of Medical Sciences, Torbat Jam, Iran

**Keywords:** Body Mass Index, Waist circumference, Women, Multi-Theory Model, Education

## Abstract

**Background:**

Considering the prevalence of overweight and abdominal obesity in middle-aged women, this study was conducted to determine the effect of empowerment program to reduce sugar consumption based on the Multi-Theory Model (MTM) on Body Mass Index (BMI) and abdominal obesity in women aged 30–60 in Joven.

**Methods:**

This quasi-experimental study (include descriptive and interventional sections) was conducted on the Joven city, Khorasan Razavi province, Iran country from October 2020 to August 2021. Sampling was performed as a multi-stage cluster. First, a descriptive study was performed among 400 women, and then 128 people who were eligible to enter the interventional phase of the study were selected. In the control group, 63 people and in the intervention group, 65 people were eligible to enter the study. The educational intervention was performed in five 60-minute sessions for groups of 12 people. The instruments included the demographic questionnaire, sugar consumption checklist and researcher-made questionnaire based on MTM constructs. Before the intervention, one, three and six months after the intervention, the questionnaire was completed by both intervention and control groups also measurement of waist circumference and BMI were performed using standard instruments. The obtained data were analyzed by SPSS 17.

**Results:**

After the educational intervention, there was a significant difference between the intervention and control groups in all the MTM constructs. Also, six months after the educational intervention, BMI, waist circumference and amount of consumption of sugary substances decreased significantly in the intervention group (p < 0.05).

**Conclusion:**

Educational intervention based on the MTM can be effective in reducing the consumption of sugary substances and shaping behaviors related to healthy lifestyle in women.

## Background

The increasing prevalence of obesity has become one of the leading causes of non-communicable diseases and mortality worldwide [1, 2]. One of the global health concerns is unhealthy eating patterns and the consequent increasing prevalence of non-communicable diseases, such as obesity and diabetes [3]. Gradual weight gain is the result of overeating sugary high-energy foods and drinks and resulting in more calories [4]. To reduce the burden of non-communicable diseases, the World Health Organization has advised countries to limit “free sugar” consumption to less than 10% of total energy intake [5]. Consumption of sugar in Iran is equal to 59 g per day and 20% more than the global average, which is 4 times higher than in the Far East [6]. The incidence of obesity varies between 18.5% and 38.3% in the United States [7]. In the UK, approximately 33% and 23% of women are overweight and obese, respectively [8]. Iran, like many developing countries, has a high prevalence of obesity and its complications. The prevalence of obesity in the Iranian adult population is about 21.7% [9].

An adverse form of obesity with serious consequences is abdominal obesity, which has increased significantly in the world over the years, and its implications in the form of non-communicable diseases have been reported in many studies [10–12]. Due to differences in biological (such as hormonal) and behavioral characteristics, women are more likely to be overweight and obese than their male counterparts [13, 14]. Several studies have shown an association between high Body Mass Index (BMI) and a high risk of maternal complications [15–17]. Lifestyle is a dynamic chain throughout human life and plays an essential role in health [18]. Women are influential people in the lifestyle and nutritional patterns of the family and because of the responsibility of life, they pay less attention to their health. On the other hand, unhealthy eating habits increase significantly in middle age and old age [19, 20]. Although much emphasis has been placed on educational strategies and behavioral interventions aimed at reducing sugar consumption in children and adolescents, but adults have not received much attention [21]. One of the most important pillars of preventive interventions is the use of theoretical framework [22]. Existing theories and models have issues such as conceptual problems, lack of sufficient predictive power, inefficiency, over-comprehensiveness, and therefore impracticality. Thus, in 2015, Sharma proposed the Multi-Theory Model (MTM) to change health behavior [23, 24]. The MTM introduces three basic constructs in explaining and predicting the onset of a behavior: Participatory Dialogue which requires a two-way communication and focuses on the pros and cons of changing health behavior; Behavioral Confidence that focuses on believing in the ability to change behavior; and Changes in Physical Environment include improved ability to acquire, accessibility, convenience, and resource availability. In addition, the MTM includes three other constructs that affect the maintenance or continuation of health behavior change: Emotional Transformation involves changing emotions and directing them to help change health behavior; Practice for Change that emphasizes active reflection and reflective behavior; and Change in Social Environment that include creating social support in the environment [24–28]. MTM was designed based on research and studies on different models of behavior prevention and modification, and its effectiveness in predicting the initiation and maintenance of behaviors has been proven [29]. This model in various studies to improve health behavior including reducing smoking [23], creating and maintaining physical activity [28], predict portion size consumption among college students [26], prediction of appropriate sleep behavior [30] and multilateral intervention protocols have been used to prevent childhood obesity [31]. The Nahar study aimed at “utilizing MTM in determining intentions to smoking cessation among smokers”, the Sharma study aimed at “applying a new theory to smoking cessation: case of MTM for health behavior change”, and the Sharma study aimed at” using MTM of health behavior change to predict water consumption instead of sugar sweetened beverages” have shown that this model is a strong theoretical framework for designing behavior change interventions [32–34]. Very few studies have been conducted on the consumption of sugary substances in all people, especially in the age group of 30–60 years, and most of the studies have been conducted on the consumption of sweet drinks. Also, few studies have been done with the multi-theory model on the consumption of sugary substances [35, 36]. Considering the prevalence of overweight and abdominal obesity in middle-aged women and the importance of this issue in their health and considering the effect of sugar consumption on overweight, this study was conducted to determine the effect of empowerment program to reduce sugar consumption based on the MTM on BMI and abdominal obesity in women.

## Methods

### Study design and sampling

This quasi-experimental study was conducted in the Joven city in Khorasan Razavi province in northeastern Iran from October 2020 to August 2021. The descriptive section sample size was calculated 400 people using the following formula and studies [37, 38] with type I error (α) 5%, 95% confidence level and considering the probability of sample loss.

$$n = \frac{{{z_{1 - \frac{{{a^{2(pq)}}}}{2}}}}}{{{{(d)}^2}}} = \frac{{{{(1.962)}^2} \times (0/5 \times 0/5)}}{{{{(0/05)}^2}}} = 384 \sim 400$$

A total of 126 persons (63 people for each group) were considered for the interventional section sample size using G-Power software according to the previous study [35], effect size 65%, type I error (α) 5%, test power 95% with considering the probability of sample loss.

In order to prevent the intervention group from contacting the control group and transferring the training content, the intervention and control groups were randomly selected from different health centers and no random allocation was done between them.

**Fig. 1:**
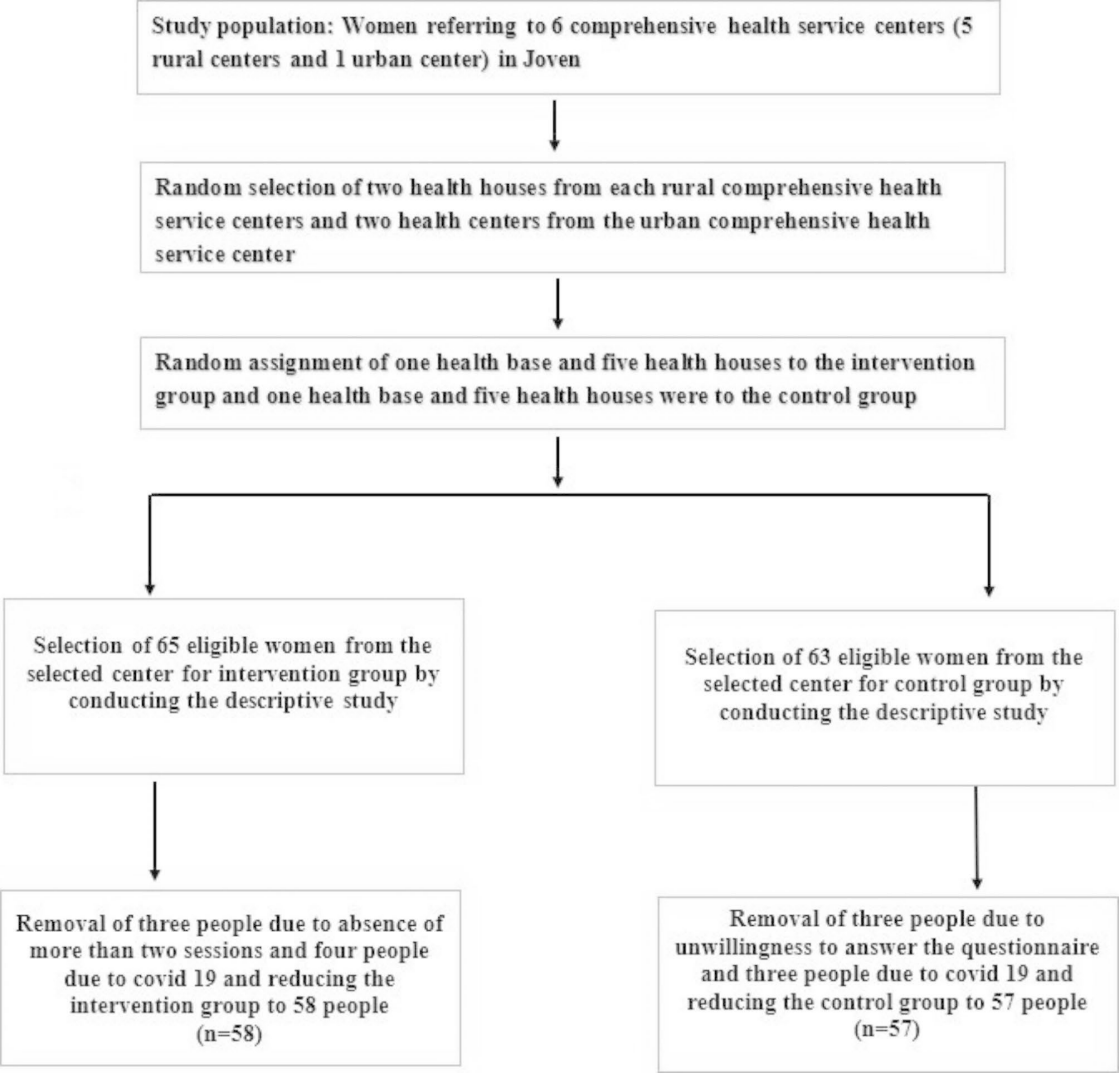
Sampling chart

Sampling was performed as a multi-stage cluster. Initially, each of the 6 comprehensive health service centers (5 rural centers and 1 urban center) covered by the Joven city was considered as a cluster. Then, from each of the 5 rural comprehensive health service centers, 2 health houses were randomly selected (to reduce cultural differences) and from the urban comprehensive health service center, 2 health bases were randomly selected so that the samples included urban and rural women. Then one health base and 5 health houses were randomly placed in the intervention group and one health base and 5 health houses in the control group. After a descriptive study, 128 women with the conditions to enter the intervention section of the study were selected. In the control group, 63 people and in the intervention group, 65 people were eligible to enter the study. Inclusion criteria for entering the intervention section according to the descriptive stage questionnaire included having excessive consumption of sugars (more than 10%), have normal results of screening tests for fasting blood sugar (FBS < 126 mg/dL) in less than a year, having a BMI higher than 25 pregnancy, not lactation, not premature menopause, being fertile, not underlying disease (thyroid, depression, diabetes, cardiovascular disease, osteoarthritis), not taking hormonal medications and completing the informed written consent form for voluntary participation in the study. Exclusion criteria included failure to cooperate with researchers in research stages, and infection or suspicion of Covid 19. The number of people in the intervention group was reduced to 58 because 3 people were removed due to absence for more than two sessions and 4 people due to Covid 19. The number of people in the control group was reduced to 57 because 3 people refused to participate in the study due to unwillingness to answer the questionnaire, and 3 people refused to continue participating in the study due to being infected with Covid-19 (Fig. 1).

### Data collection tools

The instruments used in the descriptive part of the study included a demographic questionnaire (age, education level, occupation, spouse occupation, family income, place of residence, height, weight, Waist circumference and family history of obesity; as well as questions such as the history of illness, history of medication use, and complete laboratory findings over the past year) and a sugar consumption checklist (sugar intake over the past week as 7 options: never or less than once a week, once a week, 2–4 times a week, 5–6 times a week, once a day, 2–4 times a day, 5 or more times a day). The instrument used in the intervention part of the research included a researcher-made questionnaire based on MTM constructs. To design this questionnaire, the guide for compiling MTM constructs, reviewing relevant valid texts and also the results of the descriptive part of the study were used.

In total, the questionnaire consisted of 48 questions including Participatory Dialogue in the form of 17 questions, Behavioral Confidence in the form of 9 questions, Changes in Physical Environment in the form of 6 questions, Emotional Transformation in the form of 6 questions with a 5-point Likert scale from strongly disagree to strongly agree; also Practice for Change in the form of 4 questions, Change in Social Environment in the form of 4 questions with a 5-point Likert scale with options never, rarely, sometimes, often and always and start behavioral changes with 1 question and maintain or continue the change in the form of 1 question with a 6-point Likert scale from I can completely to I cannot at all.

Qualitative and quantitative methods were used to determine the face validity of the instrument. In a qualitative evaluation of face validity, the questionnaire was given to 20 eligible women aged 30–60 years and items such as the level of difficulty in understanding phrases and words, the degree of appropriateness and optimal relationship of phrases with the dimensions of the questionnaire and ambiguities and misconceptions of the proposed phrases were examined. Then, in order to reduce and eliminate inappropriate phrases and determine the importance of each phrase, the quantitative method of “Item Impact Score” was used and the impact score higher than 1.5 was considered acceptable. Both qualitative and quantitative methods were used to determine the content validity. In the qualitative method of the questionnaire, 11 health education specialists and tool design specialists were asked to review the questionnaire based on the criteria of grammar, use of appropriate words, placement of items in the right place, proper scoring, appropriateness of selected dimensions and questions. At this stage, 16 questions were corrected and 8 questions were deleted. In determining the content validity by quantitative method, two indices of Content Validity Ratio (CVR) and Content Validity Index (CVI) were calculated. To determine the CVR, the experts of the previous stage (11 people) were asked to judge each question in relation to its content in three ways: necessary, useful or unnecessary. CVR values higher than 0.62 were accepted based on the Lawshe table. The content validity ratio was calculated to be 0.94. CVI values were determined by three criteria (relevance, simplicity, and clarity) using a four-part Likert scale for each item. The minimum acceptable value for the CVI index is 0.79 [38]. Items with a score of 0.7 to 0.79 were decided by the researcher’s judgment and renegotiated with experts. The content index of the questionnaire was calculated to be 0.92. The reliability of the questionnaire was determined using the Internal Consistency method. The questionnaire was completed by 30 eligible women aged 30–60 and internal consistency (by calculating Cronbach’s alpha) was determined (Table 1).

**Table 1:** Cronbach’s alpha values and sample questions of the questionnaire domains

Questionnaire domains		Number of questions	Cronbach’s alpha coefficients (N = 30)	Sample question
Participatory Dialogue	Pros	12	0.92	I can manage my weight if I consume less sugar
	Cons	5	0.93	I will have less energy if I consume less sugar
Behavioral Confidence		9	0.89	I’m sure I do not consume sugar, even in the absence of others
Changes in Physical Environment		6	0.71	I’m sure I can buy healthy foods instead of sugar
Emotional Transformation		6	0.73	I’m sure I can overcome the temptation to eat sugar
Practice for Change		4	0.8	I’m sure I can monitor my sugar intake by taking notes daily
Change in Social Environment		4	0.76	I’m sure my spouse will support me in reducing my sugar intake
Start Behavioral Changes		1	0.93	You are likely to reduce your sugar intake in the coming weeks
Maintain or Continue the Change		1	0.93	You are likely to consume less sugar in the next 6 months than in the past

### Educational intervention program

First, a descriptive study was performed using the sugar consumption checklist among 400 women in the age group of 30–60 years in the city after completing the written consent form by individuals. After analyzing the results of the collected information, the number of 128 people who were eligible to enter the interventional phase of the study (including having a high consumption of sugary substances more than 10%, having a body mass index higher than 25 and having had normal blood lipid, thyroid, and blood sugar tests over the past year) were identified.

After holding a briefing session on the objectives of the research and providing assurance in terms of confidentiality and completing the written consent form to participate in the research, the questionnaire based on MTM constructs was completed by the intervention and control groups. The data were entered into SPSS 17 software and the educational content was designed based on the results obtained from data analysis and using valid sources, and then the educational interventions were implemented according to the program. The educational intervention was performed in five 60-minute sessions in the intervention group using the content based on MTM constructs. Intervention methods included lectures, questions and answers, expression of vicarious experiences, emotional and physiological states, group discussion, role playing, using the technique of brainstorming and showing educational video (Table 2).

**Table 2:** The educational program for Intervention Group

Sessions	Objectives	Educational content and methods
First sessions (60 min; Five groups of approximately 12 people)	Improving women’s knowledge about sugary substances	Topics: Introduction and communication, definition of sugar substances, standard of sugar consumption, side effects of sugar consumption, importance and benefits of reducing sugar consumption Training method: Lectures with group discussion and brainstorming
Second sessions (60 min; Five groups of approximately 12 people)	Increase self-efficacy and perceived behavioral control of women to reduce sugar intake	Topics: Introduction and communication, expression of vicarious experiences along with showing videos about the side effects of sugar consumption to increase the motivation to reduce sugar consumption, building confidence in the ability to reduce sugar consumption, breaking the desired behavior into smaller and simpler units Training method: lectures, Group discussion using strategies of vicarious experiences and emotional and physiological states, playing video
Third sessions (60 min; Five groups of approximately 12 people)	Focus on emotional transformations, and guide the positive emotions of women in reducing sugar consumption	Topics: Introduction and communication, screening of videos about the side effects of sugar consumption, description of diseases that occur in order to consume sugar, list the problems to reduce sugar consumption, how to deal with potential problems in reducing sugar consumption, provide practical solutions to deal with problems Training method: Lectures, playing video, distribution of educational content through cyberspace
Fourth sessions (60 min; Five groups of approximately 12 people)	Facilitate practice for change (increase women ‘s self - monitoring of reducing sugar consumption and setting the purpose to reduce sugar consumption	Topics: Introduction and communication, record daily consumables in the notebook, providing a booklet on ways to reduce sugar consumption, check the label on food to check the amount of sugar in food, review the diet of the individual and family and introduce healthy food alternatives to unhealthy foods Training method: brainstorming, role playing
Fifth sessions (60 min; Five groups of approximately 12 people)	Facilitate change in the social environment (help and support of friends, family and health care providers to women in reducing sugar consumption)	Topics: Introduction and communication, enabling people to gain social support from friends and family to reduce sugar consumption, provide an educational booklet to guide behavior to reduce sugar consumption, emotional and informational support to help reduce sugar consumption Training method: lectures, playing video and provide the educational booklet

One, three and six months after the intervention, the questionnaire based on MTM constructs was completed by both intervention and control groups. To determine the effect of the intervention on the behavior of individuals and its outcome, measurement of Waist circumference and BMI were performed using standard instruments, before the intervention, one, three and six months after the intervention. At each stage, the participants were invited to the health centers and the questionnaires were completed by them under the supervision of a health education specialist. Anthropometric quantities were achieved by a trained physician. Height and Waist size(cm) were determined to the nearest 0.1 cm with a stadiometer and weight was measured to the nearest 0.1 kg on a portable Seca 700 (Seca, Germany). BMI was calculated as weight in kilograms divided by the square of height in meters.

### Statistical analysis

At first, we checked the normality of the variables with the Kolmogorov Smirnov test. Independent t-test was used to examine the difference between the mean of quantitative demographic variables and the main research variables in the intervention and control groups. Chi-square test was used to check the difference in the ratio of qualitative variables in the intervention and control groups. Finally, we used the Generalized Estimating Equations (GEE) test to check the mean difference of the main variables over time (Before the intervention(t1), 1 month after the intervention(t2), 3 months after the intervention(t3) and 6 months after the intervention(t4) by group (intervention and control). Then, multivariable linear regression by ENTER technique was also used to investigate the association between the start and maintain behavior with MTM constructs. The data analysis was done with SPSS 17 and the level of confidence in all the tests was considered to be 95%.

### Ethical considerations

This research was approved by the Ethics Committee of Sabzevar University of Medical Sciences, Code IR.MEDSAB.REC.1398.044. While obtaining informed consent from subjects, participants were assured that their information would remain confidential.

## Results

The average age of the studied participants in the intervention and the control group was 38.17 ± 4.86 and 39.35 ± 5.64, respectively (p = 0.46), and almost 80% participants in the intervention and control groups lived in the village (p = 0.85). On the other hand, the variables of waist size, BMI, education, occupation, spouse’s occupation, income, history of disease, history of drug use and history of maternal obesity in the intervention and control groups were not significantly difference (p > 0.05, Table 3).

**Table 3:** Comparison of demographic variables in intervention and control groups before educational intervention

Variable	Category	Intervention group (n = 58)	Control group (n = 57)	p-value
Age(yr), mean (SD)	NA	38.17 ± 4.86	39.35 ± 5.64	0.461^a^
Waist size(cm), mean (SD)	NA	97.46 ± 7.94	100.31 ± 9.58	0.085^a^
BMI(kg/m^2)^, mean (SD)	NA	33.22 ± 4.96	34.12 ± 3.88	0.587^a^
Weight (Kg), mean (SD)	NA	86.83 ± 10.78	89.42 ± 8.76	0.161
Place of residence frequency (percent)	City	12 (20.69)	11(19.3)	0.855^b^
	Village	47(79.31)	47(80.7)	
Job frequency (percent)	Employee	2(3.45)	3(5.26)	0.734^b^
	Freelance	3(5.17)	4(7.02)	
	housewife	53(91.38)	50(87.72)	
Level of Education frequency (percent)	Illiterate	5(8.62)	5(8.78)	0.784^b^
	Under diploma	21(36.21)	23(40.35)	
	Diploma	15(25.86)	17(29.82)	
	University	17(29.31)	12(21.05)	
Marital status frequency (percent)	Married	53(91.38)	55(96.5)	0.387^b^
	Not married	3(5.17)	1(1.75)	
	Other	2(3.45)	1(1.75)	
Spouse job frequency (percent)	Employee	5(8.62)	3(5.26)	0.384^b^
	Retired	2(3.45)	3(5.26)	
	Freelance	45(77.58)	42(73.69)	
	Unemployed	6(10.35)	9(15.79)	
History of disease frequency (percent)	Yes	8(13.79)	6(10.53)	0.596^b^
	No	50(86.21)	51(89.47)	
History of drug use frequency (percent)	Yes	6(10.35)	4(7.02)	0.747^b^
	No	52(89.65)	53(92.97)	
History of maternal obesity frequency (percent)	Yes	31(53.45)	27(47.37)	0.585^b^
	No	27(46.55)	30(52.63)	
History of father obesity frequency (percent)	Yes	14(24.14)	9(15.79)	0.354^b^
	No	44(75.86)	48(84.21)	
Fasting Blood Sugar(mg/dL)	< 126	52(89.65)	54(94.74)	0.491^b^
	> 126	6(10.35)	3(5.26)	

BMI: Body mass index, NA: Not applicable, a: Independent t-test, b: Chi-square test, significance level < 0.05

Table 4 shows the Comparison of MTM constructs in the intervention and control groups (before the intervention, one, three and six months after the intervention). According to the results of the GEE tests, the time, group and time*group effect are significant in all variables except Waist size(cm) (just group effect is significant) (p < 0.05), therefore, the overall average of the variables is different in 4 time periods (Time effect), the overall average difference of the variables in the test and control group is different (Group effect), and also the trend of the average difference in the test and control group is different (Time*Group effect).

According to the results of the Independent t-tests, the mean of BMI for 6 months after the intervention, in case group is (32.18 ± 4.16) lower than control group (34.15 ± 2.03) (p < 0.001).

The mean of Waist size(cm) for 3 months after the intervention, in case group is (96.87 ± 7.36) lower than control group 100.14 ± 9.31) (p < 0.001) and for 6 months after the intervention, in case group is (95.76 ± 6.84) lower than control group (99.98 ± 9.35).The mean of Participatory Dialogue (total) for 1 months after the intervention, in case group is (38.05 ± 8.65) more than control group (31.56 ± 7.59) (p < 0.001), for 3 months after the intervention, in case group is (45.24 ± 5.60) more than control group (36.31 ± 11.12) (p < 0.001) and for 3 months after the intervention, in case group is (47.32 ± 5.41) more than control group (34.84 ± 9.84) (p < 0.001).The mean of Participatory Dialogue (pros) for 1 months after the intervention, in case group is (49.86 ± 8.14) more than control group (49.68 ± 8.46) (p < 0.001), for 3 months after the intervention, in case group is (54.40 ± 4.07) more than control group (48.01 ± 7.25) (p < 0.001) and for 3 months after the intervention, in case group is (55.84 ± 3.72) more than control group (48.02 ± 4.49) (p < 0.001).The mean of Participatory Dialogue (cons) for before intervention, in case group is (12.43 ± 4.98) lower than control group (14.94 ± 4.49) (p = 0.005), for 3 months after the intervention, in case group is (9.15 ± 2.63) lower than control group (13.37 ± 5.04) (p < 0.001) and for 3 months after the intervention, in case group is (8.52 ± 2.76) more than control group (13.17 ± 4.94) (p < 0.001).The mean of Behavioral Confidence for 1 months after the intervention, in case group is (34.84 ± 5.41) more than control group (30.35 ± 5.14) (p < 0.001), for 3 months after the intervention, in case group is (38.83 ± 3.31) more than control group (31.84 ± 5.35) (p < 0.001) and for 3 months after the intervention, in case group is (38.67 ± 3.27) more than control group (31.10 ± 5.65) (p < 0.001).The mean of Changes in Physical Environment for before intervention, in case group is (21.58 ± 2.82) more than control group (20.31 ± 3.35) (p = 0.015), for 1 months after the intervention, in case group is (23.78 ± 2.52) more than control group (21.67 ± 3.33) (p = 0.002), for 3 months after the intervention, in case group is (25.86 ± 2.42) more than control group (22.14 ± 2.95) (p < 0.001) and for 3 months after the intervention, in case group is (26.79 ± 2.28) more than control group (22.01 ± 3.12) (p < 0.001).The mean of Emotional Transformation for 1 months after the intervention, in case group is (20.47 ± 3.40) more than control group (18.56 ± 3.90) (p = 0.004), for 3 months after the intervention, in case group is (22.24 ± 2.52) more than control group (19.31 ± 3.64) (p < 0.001) and for 3 months after the intervention, in case group is (22.71 ± 2.49) more than control group (18.67 ± 3.72) (p < 0.001).The mean of Practice for Change for 1 months after the intervention, in case group is (15.53 ± 1.85) more than control group (11.00 ± 2.58) (p < 0.001), for 3 months after the intervention, in case group is (17.60 ± 1.73) more than control group (11.42 ± 2.85) (p < 0.001) and for 3 months after the intervention, in case group is (18.19 ± 1.81) more than control group (11.44 ± 2.71) (p < 0.001).The mean of Change in Social Environment for 1 months after the intervention, in case group is (16.14 ± 1.89) more than control group (13.95 ± 2.76) (p < 0.001), for 3 months after the intervention, in case group is (17.34 ± 1.50) more than control group (14.26 ± 2.53) (p < 0.001) and for 3 months after the intervention, in case group is (18.12 ± 1.46) more than control group (14.40 ± 2.12) (p < 0.001).The mean of Start Behavioral Changes for 1 months after the intervention, in case group is (4.77 ± 0.80) more than control group (3.91 ± 1.04) (p < 0.001), for 3 months after the intervention, in case group is (5.29 ± 0.070) more than control group (4.33 ± 1.17) (p < 0.001) and for 3 months after the intervention, in case group is (5.57 ± 0.62) more than control group (4.47 ± 1.17) (p < 0.001). The mean of Maintain or Continue the Change for 3 months after the intervention, in case group is (5.36 ± 0.78) more than control group (4.42 ± 1.21) (p < 0.001) and for 3 months after the intervention, in case group is (5.55 ± 0.73) more than control group (4.40 ± 1.21) (p < 0.001)(Table 4).

**Table 4:** Comparison of MTM constructs in the intervention and control groups (before the intervention, one, three and six months after the intervention)

	Group	Mean ± SD				P-value(b)	P-value(c)	P-value(d)
		Before the intervention(t1)	1 month after the intervention(t2)	3 months after the intervention(t3)	6 months after the intervention(t4)	Time	Group	Time* Group (Interaction)
BMI(kg/m2)	Control	34.12 ± 3.88	34.78 ± 3.31	33.98 ± 2.21	34.15 ± 2.03	**< 0.001**	**0.045**	**0.017**
	Intervention	33.22 ± 4.96	33.27 ± 4.96	32.95 ± 4.93	32.18 ± 4.16			
	p-value(a)	**0.587**	**0.080**	**0.067**	**< 0.001**			
Waist size(cm)	Control	100.31 ± 9.58	100.29 ± 9.54	100.14 ± 9.31	99.98 ± 9.35	**0.831**	**0.014**	**0.069**
	Intervention	97.46 ± 7.94	97.38 ± 7.67	96.87 ± 7.36	95.76 ± 6.84			
	p-value(a)	**0.085**	**0.082**	**0.045**	**0.010**			
Weight (Kg)	Control	89.42 ± 8.76	89.48 ± 8.78	88.57 ± 8.68	89.48 ± 8.78	**< 0.001**	**0.038**	**0.014**
	Intervention	86.83 ± 10.78	86.79 ± 10.73	85.53 ± 10.64	86.79 ± 10.73			
	p-value(a)	**0.161**	**0.145**	**0.09**	**0.023**			
Participatory Dialogue (total)	Control	33.70 ± 7.69	31.56 ± 7.59	36.31 ± 11.12	34.84 ± 9.84	**< 0.001**	**< 0.001**	**< 0.001**
	Intervention	36.83 ± 6.76	38.05 ± 8.65	45.24 ± 5.60	47.32 ± 5.41			
	p-value(a)	**0.095**	**< 0.001**	**< 0.001**	**< 0.001**			
Participatory Dialogue (pros)	Control	36 ± 7.15	49.68 ± 8.46	48.01 ± 7.25	48.02 ± 4.49	**< 0.001**	**< 0.001**	**< 0.001**
	Intervention	49.25 ± 4.53	49.86 ± 8.14	54.40 ± 4.07	55.84 ± 3.72			
	p-value(a)	**0.558**	**< 0.001**	**< 0.001**	**< 0.001**			
Participatory Dialogue (cons)	Control	14.94 ± 4.49	13.05 ± 4.76	13.37 ± 5.04	13.17 ± 4.94	**< 0.001**	**< 0.001**	**< 0.001**
	Intervention	12.43 ± 4.98	11.81 ± 3.64	9.15 ± 2.63	8.52 ± 2.76			
	p-value(a)	**0.005**	**0.112**	**< 0.001**	**< 0.001**			
Behavioral Confidence	Control	31.82 ± 5.28	30.35 ± 5.14	31.84 ± 5.35	31.10 ± 5.65	**< 0.001**	**< 0.001**	**< 0.001**
	Intervention	31.90 ± 3.41	34.84 ± 5.41	38.83 ± 3.31	38.67 ± 3.27			
	p-value(a)	**0.623**	**< 0.001**	**< 0.001**	**< 0.001**			
Changes in Physical Environment	Control	20.31 ± 3.35	21.67 ± 3.33	22.14 ± 2.95	22.01 ± 3.12	**< 0.001**	**< 0.001**	**< 0.001**
	Intervention	21.58 ± 2.82	23.78 ± 2.52	25.86 ± 2.42	26.79 ± 2.28			
	p-value(a)	**0.015**	**0.002**	**< 0.001**	**< 0.001**			
Emotional Transformation	Control	19.70 ± 3.63	18.56 ± 3.90	19.31 ± 3.64	18.67 ± 3.72	**0.002**	**< 0.001**	**< 0.001**
	Intervention	20.10 ± 2.52	20.47 ± 3.40	22.24 ± 2.52	22.71 ± 2.49			
	p-value(a)	**0.416**	**0.004**	**< 0.001**	**< 0.001**			
Practice for Change	Control	11.89 ± 2.46	11.00 ± 2.58	11.42 ± 2.85	11.44 ± 2.71	**< 0.001**	**< 0.001**	**< 0.001**
	Intervention	12.10 ± 1.90	15.53 ± 1.85	17.60 ± 1.73	18.19 ± 1.81			
	p-value(a)	**0.547**	**< 0.001**	**< 0.001**	**< 0.001**			
Change in Social Environment	Control	13.79 ± 2.56	13.95 ± 2.76	14.26 ± 2.53	14.40 ± 2.12	**< 0.001**	**< 0.001**	**< 0.001**
	Intervention	13.79 ± 2.28	16.14 ± 1.89	17.34 ± 1.50	18.12 ± 1.46			
	p-value(a)	**0.199**	**< 0.001**	**< 0.001**	**< 0.001**			
Start Behavioral Changes	Control	3.77 ± 1.26	3.91 ± 1.04	4.33 ± 1.17	4.47 ± 1.17	**< 0.001**	**< 0.001**	**< 0.001**
	Intervention	3.87 ± 0.96	4.77 ± 0.80	5.29 ± 0.070	5.57 ± 0.62			
	p-value(a)	**0.578**	**< 0.001**	**< 0.001**	**< 0.001**			
Maintain or Continue the Change	Control	4.24 ± 1.36	4.21 ± 1.28	4.42 ± 1.21	4.40 ± 1.21	**< 0.001**	**0.004**	**< 0.001**
	Intervention	3.88 ± 1.28	4.48 ± 1.08	5.36 ± 0.78	5.55 ± 0.73			
	p-value(a)	**0.067**	**0.230**	**< 0.001**	**< 0.001**			

a) Independent t-test

b) Time effect in Generalized Estimating Equation (GEE)

c) Group effect in Generalized Estimating Equation (GEE)

d) Time*Group effect (Interaction) in Generalized Estimating Equation (GEE)

Table 5 shows Comparison of the Consumption of sugary substances in the intervention and control groups (before the intervention, one, three and six months after the intervention). According to the results of the GEE tests, the time, group and time*group effect are significant in all variables except sugar (just group effect is not significant) (p < 0.05), therefore, the overall average of the variables is different in 4 time periods (Time effect), the overall average difference of the variables in the test and control group is different (Group effect), and also the trend of the average difference in the test and control group is different (Time*Group effect). According to the results of the Independent t-tests, the mean of sugar Loaf for 1 months after the intervention, in case group is (4.89 ± 1.42) lower than control group (5.40 ± 1.51) (p = 0.011), for 3 months after the intervention, in case group is (2.65 ± 1.85) lower than control group (5.54 ± 1.50) (p = 0.002) and for 3 months after the intervention, in case group is (2.43 ± 1.52) more than control group (5.54 ± 1.51) (p < 0.001). The mean of sugar for 1 months after the intervention, in case group is (1.21 ± 0.67) lower than control group (1.68 ± 1.08) (p = 0.001), for 3 months after the intervention, in case group is (1.12 ± 0.42) lower than control group (1.70 ± 1.22) (p < 0.001) and for 3 months after the intervention, in case group is (1.10 ± 0.41) more than control group (1.70 ± 1.22) (p < 0.001). The mean of Chocolate for 1 months after the intervention, in case group is (2.19 ± 1.16) lower than control group (3.29 ± 1.43) (p < 0.001), for 3 months after the intervention, in case group is (1.31 ± 0.68) lower than control group (3.39 ± 1.47) (p < 0.001) and for 3 months after the intervention, in case group is (1.22 ± 0.46) more than control group (3.37 ± 1.48) (p < 0.001). The mean of jam for 3 months after the intervention, in case group is (1.33 ± 0.57) lower than control group (2.12 ± 1.16) (p < 0.001) and for 3 months after the intervention, in case group is (1.29 ± 0.53) more than control group (2.10 ± 1.17) (p < 0.001). The mean of candy for 1 months after the intervention, in case group is (1.10 ± 0.41) lower than control group (1.33 ± 0.72) (p = 0.018), for 3 months after the intervention, in case group is (1.03 ± 0.18) lower than control group (1.33 ± 0.72) (p = 0.002) and for 3 months after the intervention, in case group is (1.02 ± 0.13) more than control group (1.33 ± 0.72) (p = 0.001)(Table 5).

**Table 5:** Comparison of the Consumption of sugary substances in the intervention and control groups (before the intervention, one, three and six months after the intervention)

	Group	Mean ± SD				P-value(b)	P-value(c)	P-value(d)
		Before the intervention(t1)	1 month after the intervention(t2)	3 months after the intervention(t3)	6 months after the intervention(t4)	Time	Group	Time* Group (Interaction)
sugar Loaf (Time/week)	Control	5.42 ± 1.56	5.40 ± 1.51	5.54 ± 1.50	5.54 ± 1.51	**< 0.001**	**< 0.001**	**< 0.001**
	Intervention	5.94 ± 1.02	4.89 ± 1.42	2.65 ± 1.85	2.43 ± 1.52			
	p-value(a)	**0.100**	**0.011**	**< 0.001**	**< 0.001**			
Sugar (Time/week)	Control	1.74 ± 1.16	1.68 ± 1.08	1.70 ± 1.22	1.70 ± 1.22	**0.009**	**0.344**	**< 0.001**
	Intervention	1.56 ± 1.16	1.21 ± 0.67	1.12 ± 0.42	1.10 ± 0.41			
	p-value(a)	**0.356**	**0.002**	**0.001**	**< 0.001**			
Chocolate (Time/week)	Control	3.33 ± 1.47	3.29 ± 1.43	3.39 ± 1.47	3.37 ± 1.48	**< 0.001**	**< 0.001**	**< 0.001**
	Intervention	3.29 ± 1.49	2.19 ± 1.16	1.31 ± 0.68	1.22 ± 0.46			
	p-value(a)	**0.977**	**< 0.001**	**< 0.001**	**< 0.001**			
Soft drinks (Time/week)	Control	2.63 ± 1.43	2.63 ± 1.40	2.77 ± 1.38	2.74 ± 1.40	**< 0.001**	**< 0.001**	**< 0.001**
	Intervention	2.57 ± 1.35	1.88 ± 0.84	1.10 ± 0.31	1.07 ± 0.25			
	p-value(a)	**0.812**	**0.003**	**< 0.001**	**< 0.001**			
jam (Time/week)	Control	2.12 ± 1.16	2.12 ± 1.16	2.12 ± 1.16	2.10 ± 1.17	**< 0.001**	**0.014**	**< 0.001**
	Intervention	2.22 ± 1.20	1.86 ± 0.88	1.33 ± 0.57	1.29 ± 0.53			
	p-value(a)	**0.594**	**0.323**	**< 0.001**	**< 0.001**			
candy (Time/week)	Control	1.33 ± 0.72	1.33 ± 0.72	1.33 ± 0.72	1.33 ± 0.72	**0.021**	**0.027**	**0.021**
	Intervention	1.28 ± 0.72	1.10 ± 0.41	1.03 ± 0.18	1.02 ± 0.13			
	p-value(a)	**0.458**	**0.018**	**0.002**	**0.001**			

a) Independent t-test

b) Time effect in Generalized Estimating Equation (GEE)

c) Group effect in Generalized Estimating Equation (GEE)

d) Time*Group effect (Interaction) in Generalized Estimating Equation (GEE)

The results of multivariable linear regression showed that for one unit of change in the score of behavioral confidence, the average the start behavior score increases to 0.308(p-0.001). Furthermore, for one unit of change in the score of practice for change and change in social environment, the average the maintain behavior score increases to 0.233(p = 0.013), and 0.242(p = 0.006), respectively (Tables 6 and 7).

**Table 6:** Factors affecting of start Behavior using multivariable linear regression model. Model based on 114 observations, adjusted R-squared = 8.08%, p = 0.004

Variable	B	SE	Standardized Coefficients Beta	P-value
Participatory Dialogue (total)	0.014	0.013	0.108	0.301
Behavioral Confidence	0.078	0.023	0.308	0.001
Perceived external and internal rewards	-0.005	0.034	-0.014	0.892

**Table 7:** Factors affecting of maintain Behavior using multivariable linear regression model. Model based on 114 observations, adjusted R-squared = 20.1%, p = < 0.001

Variable	B	SE	Standardized Coefficients Beta	P-value
Emotional Transformation	0.079	0.040	0.184	0.052
Practice for Change	0.142	0.057	0.233	0.013
Change in Social Environment	0.134	0.048	0.242	0.006

## Discussion

The aim of this study was to determine the effect of empowerment program to reduce sugar consumption based on the MTM on BMI and abdominal obesity in women aged 30–60 in Joven. Based on the results, the behavioral confidence construct had the greatest impact on the behavioral intention to reduce the consumption of sugary substances. In the studies of Nahar [39] and Hayes [40], behavioral confidence was recognized as a better predictor of behavioral intention. According to this finding, women’s belief in their abilities before taking action is one of the most important factors that determine the formation of the intention to perform the behavior. In the present study, MTM constructs predicted 10.7% of the variance of behavioral intention; While other studies have shown a spectrum between 40.8 and 26% for predicting behavior intention [28, 39, 41]. The low rate of intention prediction in the present study may be due to the fact that the women studied were selected from people who consumed sugary substances; While other studies, the target group has been selected from the entire society.

After the implementation of the educational intervention, the scores of participatory dialogue, behavioral confidence, change in the physical environment, emotional transformation, practice for change and change in the social environment increased in the intervention group compared to the control group. These results show the effectiveness of the educational intervention in increasing knowledge, understanding the risks and consequences of consuming more than the standard of sugary substances. Also, behavioral confidence, self-confidence and self-efficacy, individual motivation, focus on reducing the consumption of sugary substances and monitoring and managing the consumption pattern of sugary substances, and social support for reducing the consumption of sugary substances have been improved. These findings are consistent with the results of studies by Bashirian [23] and Brown [42]. The educational intervention had a significant effect on improving the participatory dialogue construct. This construct is derived from the perceived benefits and barriers of the health belief model and the decisional balance of the transtheoretical model [24]. Studies by Nahar [39], Sharma [32] and Hayes [40] showed that participatory dialogue is an important predictor of behavior initiation.

According to the findings of the study, the educational intervention improved the behavioral confidence construct, which is consistent with the results of the study by Bashirian [23] and Brown [42]. The behavioral confidence construct is derived from Bandura’s self-efficacy and perceived behavioral control of the theory of planned behavior [33]. In the studies of Yoshani [43], Sharma [27, 44] and Williams [41], the behavioral confidence construct was identified as the most important and strongest predictor of the initiation of behavior, which confirms the results of the present study. Women’s belief and focus on their abilities before starting to reduce the consumption of sugary substances is one of the most important determining factors that show whether women achieve the intention of doing the activity or not. Increasing self-regulation skills, such as behavioral control over the consumption of sugary substances, helps women make healthier decisions. Therefore, promoting behavioral confidence should be an integral part of health promotion efforts.

In this study, there was a significant difference in the changes in physical environment in the intervention group before and after the educational intervention, but no such difference was observed in the control group. These results are not consistent with the studies of Hayes [40], Brown [42] and Bashirian [23]. It seems that media advertisements, easy access to sugary substances, low price of sugary products compared to healthier foods are effective environmental factors in the high consumption of these substances. In order to control environmental changes, health policies, laws and decisions must be adopted at high levels.

Based on the results of the emotional transformation construct before and after the intervention, it improved significantly in the intervention group compared to the control group, which is consistent with the findings of Bashirian [23] and Hayes [40] studies. As much as people have stronger motivation and more positive feelings about reducing the consumption of sugary substances and can manage their emotions better, they will be able to reduce the consumption of these substances.

Based on the results, the practice for change construct before and after the intervention increased significantly in the intervention group compared to the control group. The findings of the present study are consistent with Brown’s study [42]. People who have more control over their behavior and focus on their abilities are better able to change their behavior. In addition, the relationship was observed between this construct and BMI, it should be said that people who monitor and manage their nutrition more can manage their weight and lead a healthier life.

Based on the results of the research, the change in social environment construct before and after the intervention significantly improved in the intervention group compared to the control group, which is consistent with the findings of Brown’s study [42] and not consistent with the results of Bashirian’s study [23]. It seems that in order to modify the change in social environment construct, the educational intervention should be designed and implemented considering the social factors affecting the behavior. Deliens’ study showed that there is a positive relationship between family norms and rules and consumption of energy drinks [45]. Changes in the physical environment require access and availability, price and purchasing power. Han’s study showed that social media as well as social determinants can have a significant effect on the choice of food and beverages [46].

The results showed that the consumption of sugary substances in the intervention group significantly decreased compared to the control group as a result of the educational intervention (especially 3 and 6 months after the educational intervention). This finding is consistent with the results of the studies of Hayes [40], Brown [42] and Crithley [47]. These results confirm the effectiveness of the MTM in changing behavior because the MTM is a complete, comprehensive and logical model that considers all behavioral factors that lead to behavior change.

The results of the study showed that waist circumference and BMI in the intervention group compared to the control group were significantly reduced six months after the educational intervention, and this difference was also observed for waist circumference three months after the educational intervention. This finding is consistent with the results of Berkey [48] and Schulze [49] studies who showed that there is a positive relationship between the consumption of sugary substances and weight gain and greater risk of obesity over time. These results show that the control of overweight in adults requires effective interventions to reduce the consumption of sugary substances and eliminate malnutrition.

One of the limitations of the study was the condition of the Covid-19 epidemic, which created problems in collecting data (non-cooperation of people due to the fear of transmission of Covid-19) and holding training sessions. Also, the impossibility of organizing training sessions for the target group in one place due to geographical dispersion and the need to hold multiple training sessions was another limitation of this research. Another limitation is that the study was conducted on women between the ages of 30 and 60, as some women are postmenopausal and have increased waist circumference due to hormonal and estrogen deprivation and not consumption of sugary substance.

## Conclusion

The use of MTM led to a deep understanding of the risks, consequences and belief in the abilities, and ultimately reduced the consumption of sugary substances and weight loss of people. This study provides practical solutions for designing and implementing educational programs and it seems that conducting research in this field and conducting interventions by adding suitable constructs or theories and its intervention in other population groups can be a prelude to further research with the aim of modifying the food pattern of the entire society.

## Data Availability

The datasets used and/or analysed during the current study are public data available from the corresponding author request.
